# A New Strategy for the Regulation of Neuroinflammation: Exosomes Derived from Mesenchymal Stem Cells

**DOI:** 10.1007/s10571-024-01460-x

**Published:** 2024-02-19

**Authors:** Ying Ge, Jingjing Wu, Li Zhang, Nanqu Huang, Yong Luo

**Affiliations:** 1 Department of Neurology, Third Affiliated Hospital of Zunyi Medical University (The First People’s Hospital of Zunyi), Zunyi, Guizhou China; 2 Medical College of Soochow University, Suzhou, Jiangsu China; 3 National Drug Clinical Trial Institution, Third Affiliated Hospital of Zunyi Medical University (The First People’s Hospital of Zunyi), Zunyi, Guizhou China

**Keywords:** Mesenchymal stem cells, Exosomes, Neuroinflammation, Glial cells, Signaling pathway

## Abstract

Neuroinflammation is an important pathogenesis of neurological diseases and causes a series of physiopathological changes, such as abnormal activation of glial cells, neuronal degeneration and death, and disruption of the blood‒brain barrier. Therefore, modulating inflammation may be an important therapeutic tool for treating neurological diseases. Mesenchymal stem cells (MSCs), as pluripotent stem cells, have great therapeutic potential for neurological diseases due to their regenerative ability, immunity, and ability to regulate inflammation. However, recent studies have shown that MSC-derived exosomes (MSC-Exos) play a major role in this process and play a key role in neuroprotection by regulating neuroglia. This review summarizes the recent progress made in regulating neuroinflammation by focusing on the mechanisms by which MSC-Exos are involved in the regulation of glial cells through signaling pathways such as the TLR, NF-κB, MAPK, STAT, and NLRP3 pathways to provide some references for subsequent research and therapy.

**Graphical Abstract:**

Exosomes derived from MSCs exhibit neuroprotective effects by regulating signaling pathways and mitigating neuroinflammation triggered by glial cells.

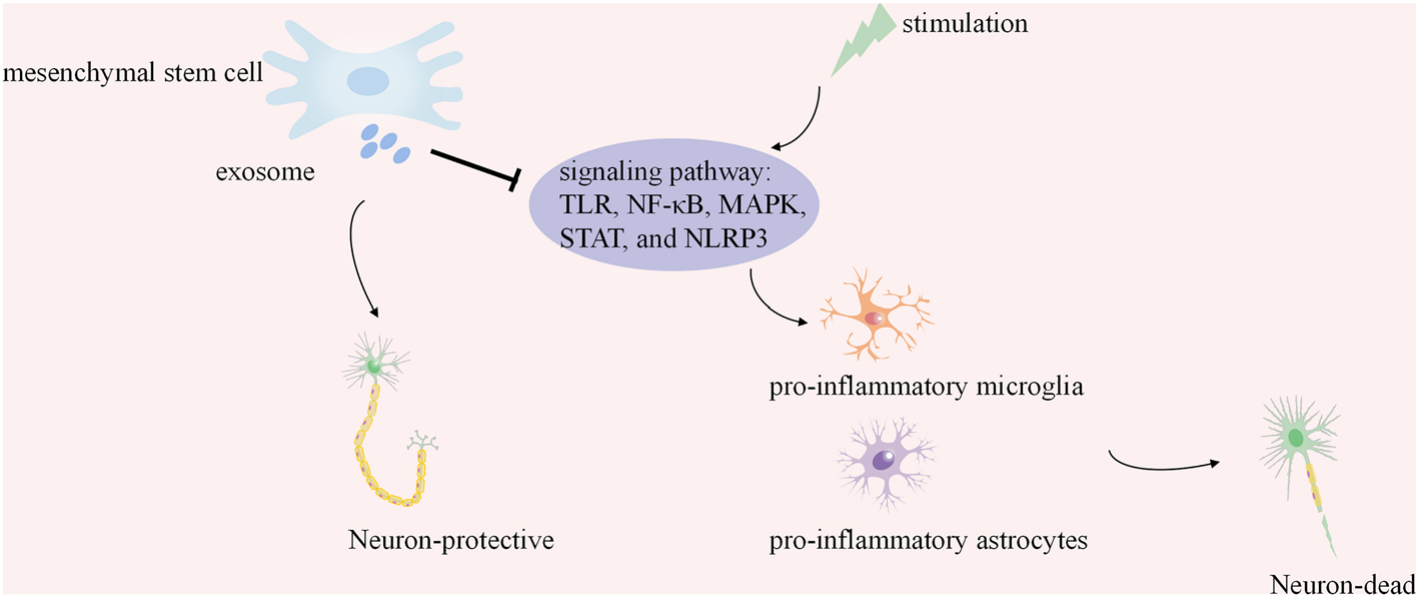

## Introduction

Neuroinflammation refers to the inflammatory response that arises when central nervous system (CNS) damage is induced by either endogenous or exogenous stimuli and is accompanied by the activation of neuroglia, particularly microglia and astrocytes (Kwon and Koh 2020; Leng and Edison 2021). In the initial phase of CNS injury, inflammation exerts a protective effect by eliminating harmful substances. However, a sustained inflammatory response continuously stimulates neuroglia, releasing inflammatory factors, and mediators. This activation prompts neuronal degeneration, impairs the blood‒brain barrier (BBB), and exacerbates brain damage via various mechanisms (Liddelow and Barres 2017; Rodriguez-Gomez et al. 2020). Neuroinflammation significantly contributes to the initiation and progression of neurodegenerative conditions and acute CNS ailments (Stephenson et al. 2018; Novoa et al. 2022; Liu et al. 2022b). Consequently, targeting neuroinflammation has emerged as a promising intervention strategy.

MSCs are pluripotent stem cells that can be isolated from various tissues, such as the umbilical cord, placenta, and bone marrow; they have the ability to undergo osteogenic, lipogenic, and chondrogenic differentiation. In recent years, MSCs have attracted much attention for use in cell therapy (Levy et al. 2020). MSCs exhibit a regulatory influence on the immune response by dispensing anti-inflammatory mediators, cytokines, and immunosuppressive factors (da Silva Meirelles et al. 2006; Shi et al. 2018). MSCs, known to inhibit neuroinflammation, actively stimulate neuronal differentiation and promote neural axon growth; they also enhance damaged nerve functions (Skok 2021; Huang et al. 2022; Bagheri-Mohammadi 2021b; Ba et al. 2022). Although MSCs are known to possess therapeutic effects, these effects are believed to be primarily induced through paracrine mechanisms, and sufficient evidence supports this claim (Ha et al. 2020). Extracellular vesicles secreted by MSCs possess a bilayer lipid membrane structure and are termed MSC-Exos (Harrell et al. 2019; Qiu et al. 2019; Palmulli and van Niel 2018). These vesicles contain proteins, lipids, and nucleic acids and can be used for tissue regeneration, immunomodulation, and inflammation modulation. MSC-Exos also play a vital role in cellular transmission (Tang et al. 2021). The significant inflammatory regulatory capacity of MSC-Exos has garnered considerable interest from researchers investigating neurological disorders (Losurdo et al. 2020).

## Glial Cells and Neuroinflammation

Neuroglia, pivotal in the CNS, include microglia, astrocytes, and oligodendrocytes. These cells actively participate in the immune response within the CNS, fostering neuronal nourishment and ensuring synaptic homeostasis (Schirmer et al. 2021; Liu et al. 2023). The role of microglia and astrocytes in neuroinflammation is a subject of growing interest (Hashioka et al. 2021).

Microglia, which are derived from the embryonic yolk sac, are innate immune cells that dominate the CNS (Rodriguez-Gomez et al. 2020; Bagheri-Mohammadi 2021a). These immune cells, which dwell in the CNS, play vital roles in pathogen defense and damage repair (Subhramanyam et al. 2019). Microglia, which are indispensable for maintaining CNS homeostasis, are activated by diverse pathological stimuli. This activation gives rise to two distinct types of M1 macrophages: classical M1 macrophages and selective M2 macrophages. Proinflammatory cytokines, chemokines, and neurotoxic factors such as tumor necrosis factor-alpha (TNF-α), nitric oxide (NO), prostaglandin E2 (PGE2), interleukin-1β (IL-1β), and IL-6, which are generally secreted by M1 microglia, contribute to damage of the CNS (Zavatti et al. 2022). M2 macrophages secrete anti-inflammatory and neuroprotective factors such as IL-4, IL-10, arginase-1 (Arg-1), and chitinase 3-like 3 (Ym1), which promote nerve repair and regeneration to maintain CNS homeostasis (Zong et al. 2021). Chronically activated M1 macrophages lead to the excessive release of inflammatory mediators, intensifying neuroinflammation and exacerbating neuronal damage (Guo et al. 2022; Cowan and Petri 2018); this highlights the importance of understanding the balance between M1 and M2 microglia. Modulating microglia could thus serve as a potent intervention method to regulate neuroinflammation.

Astrocytes, abundant in the brain, are instrumental in numerous physiological processes. These processes include blood flow regulation, BBB preservation, synaptogenesis facilitation, CNS homeostasis maintenance, and neuronal function regulation (Giovannoni and Quintana 2020). Astrocytes, which share similarities with microglia, serve dual functions as proinflammatory and neuroprotective agents. When exposed to constant pathological stimuli, these cells secrete proinflammatory cytokines such as IL-1β and TNF-α. Consequently, this action elevates reactive oxygen species (ROS) production, and this escalation leads to neurodegeneration (Hasel and Liddelow 2021; Linnerbauer et al. 2020). The release of inflammatory factors by activated microglia can trigger proinflammatory astrocytes, resulting in secondary inflammatory responses (Liddelow et al. 2017). Astrocytes, which possess neuroprotective traits, generate anti-inflammatory cytokines such as IL-4 and IL-10, contributing to nerve regeneration (Li et al. 2022b). The role of neuroinflammation mediated by astrocytes in CNS disorders is significant and cannot be overlooked.

**Fig. 1:**
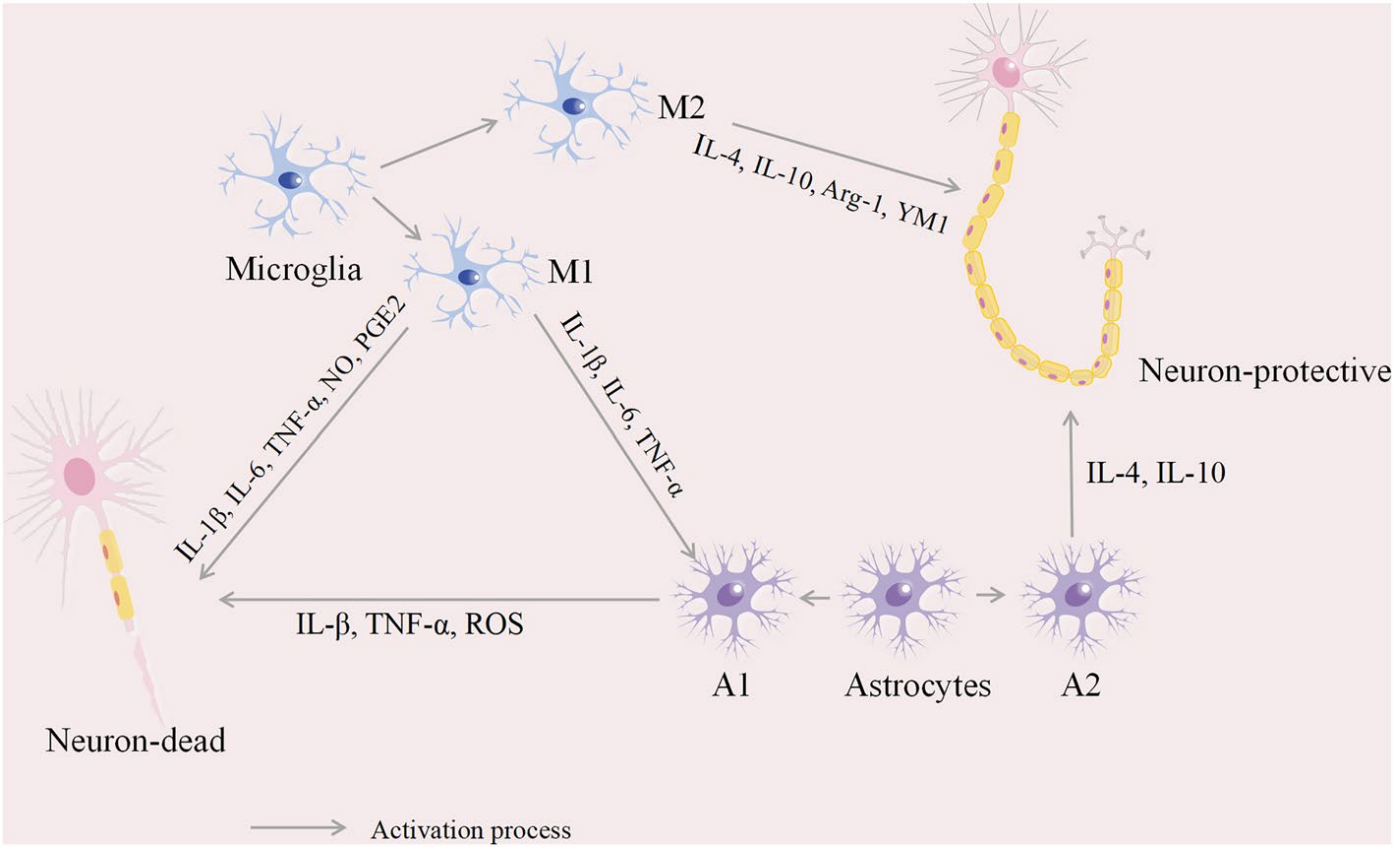
Under neuroinflammation, microglia and astrocytes are activated, releasing inflammatory factors and chemokines that lead to neuronal damage

Despite being distinct cell types, microglia and astrocytes are interconnected in their response to CNS injury; they participate in complex mechanisms that regulate neuroinflammation, and their role in the CNS is double-edged (Rueda-Carrasco et al. 2021). It is crucial to regulate glial cells and exert a protective effect on them (Fig. 1).

## The Role of MSC-Exos in CNS Disorders

CNS studies revealed that MSC-Exos exhibit neuroprotective effects by harnessing their ability to regulate inflammation, adjust neuroglial activity, and boost the functionality of damaged neural tissue (Jin et al. 2021; Liu et al. 2022a; Cui et al. 2022). As a promising, innovative therapeutic tool, MSC-Exos can be a potential game changer for neurological disorders, offering fresh hope to the medical community (Joo et al. 2020; Guo et al. 2020) (Table 1).

**Table 1:** Exosomes derived from MSCs exert neuroprotective effects, primarily by suppressing glial cell activation and regulating neuroinflammation

Disease	Biological sample	Main findings	References
AD	C57BL/6 mice	MSC-Exos improve AD-like behavioral performance, connected to its role in modulating glial activation, hippocampal neuroinflammation, and BDNF-related neuropathological changes	Liu et al. (2022a)
	APP/PS1 mice	MSC-Exos attenuate Aβ-induced neuroinflammation by inhibiting NF-κB and the STAT3 signaling pathway	Ding et al. (2018); Nakano et al. (2020); Cui et al. (2018)
	BV2 cells and PC12 cells	MSC-Exos inhibit Aβ-induced inflammatory response in microglia and protect neuronal PC12 cells	Kaniowska et al. (2022); Zhai et al. (2021)
PD	rat	MSC-Exos ameliorate neuroinflammation, reduce α-synuclein, and improve dyskinesia in rats	Chen et al. (2020c)
	SH-SY5Y cells	MSC-Exos effectively mitigates neuroinflammation, oxidative stress, and apoptosis in MPP(+)-induced Parkinson’s disease model	Li et al. (2019)
	C57BL/6 J mice and MN9D cells	MSC-Exos inhibit NLRP3 and CKD5, suppress the inflammatory response, and reduce apoptosis of dopaminergic neurons to improve dyskinesia	Li et al. (2021)
TBI	C57BL/6 mice	MSC-Exos inhibit early neuroinflammation in TBI mice by modulating microglia polarization to exert neuroprotective function	Ni et al. (2019)
	rat	Inhibition of astrocyte activation by MSC-Exos improves sensorimotor and cognitive functions in TBI rats and is time-dependent with MSC-Exo	Zhang et al. (2020)
	rat and BV2 cells	MSC-Exos inhibit HMGB1/NF-κB and/or TLR4/NFκB and MAPK signaling pathways, reducing microglia overactivation-mediated neuroinflammation	Xu et al. (2020); Thomi et al. (2019)
SE	C57BL/6 mice	MSC-Exos improve cognition in sustained epilepsy mice by modulating glial cell activation and attenuating hippocampal inflammation	Liu et al. (2021a); Long et al. (2017)
tMCAO	C57BL/6 mice	MSC-Exos inhibit microglia activation and reduce neutrophil factor and chemokine accumulation	Pathipati et al. (2021)
ALS	SOD1^G93A^ mice and astrocytes	Important mechanisms by which MSC-Exos improve prognosis in ALS are associated with inhibition of MAPK11-involved astrocyte inflammation and enhancement of antioxidant capacity	Provenzano et al. (2022)
MS	SJL/J mice	MSC-Exos modulates microglia activation in MS with immunomodulatory capacity, reduces Th1 and Th17 expression, improves motility, and promotes myelin regeneration	Laso-García et al. (2018)

### MSC-Exos and Alzheimer’s Disease

Slow progressive memory loss and cognitive impairment are the main clinical symptoms of Alzheimer’s disease (AD) (Monteiro et al. 2023). The pathological signatures of this disease typically involve excessive accumulation of extracellular Aβ and neurofibrillary tangles (NFTs) (Ratan et al. 2023; Ba et al. 2022). Glial-induced neuroinflammation has been found to significantly contribute to the acceleration of Aβ accumulation (Singh 2022; Huang et al. 2019). In contrast, MSC-Exos inhibited microglial and astrocyte activation, thereby reducing hippocampal inflammation and Aβ and tau deposits. Furthermore, it enhances synaptic function and increases brain-derived neurotrophic factor (BDNF), effectively mitigating cognitive dysfunction in AD mice (Liu et al. 2022a). In mice with amyloid precursor protein/progerin 1 (APP/PS1) mutations, MSC-Exos enhanced YM-1 and Arg-1 expression. This improvement was accompanied by superior spatial learning and memory function during water maze testing (Ding et al. 2018). MSC-Exos significantly decreased Aβ deposition in the brains of APP/PS1 mice, which is consistent with previous observations (Ding et al. 2018). MSC-Exos significantly attenuated Aβ-induced neuroinflammation by inhibiting the nuclear factor kappa B (NF-κB) signaling pathway and suppressing signal transducer and activator of transcription 3 (STAT3). This intervention also markedly improved the neurological function and locomotor ability of AD mice (Nakano et al. 2020; Cui et al. 2018). In vitro investigations demonstrated that MSC-Exos suppress the increase in proinflammatory agents such as TNF-α and NO induced by Aβ aggregation (Kaniowska et al. 2022). Furthermore, MSC-Exos protect Aβ-induced PC12 cells by reducing inflammatory factor release and attenuating PC12 cell apoptosis; this occurs through the inhibition of the nucleotide-binding oligomerization domain, leucine-rich repeat, pyrin domain-containing 3 (NLRP3), and caspase-1 (Zhai et al. 2021). Studies indicate that MSC-Exos can regulate glial cells, thereby modulating neuroinflammatory responses in AD.

### MSC-Exos and Parkinson’s Disease

PD, a neurodegenerative disorder, is the second most common disorder. Dopamine depletion is characterized by the degeneration of dopaminergic neurons, which leads to motor and nonmotor impairments (Jankovic and Tan 2020). PD has a complex pathogenesis, often caused by neuroinflammation (Wang et al. 2022). MSC-Exos play a significant role in PD treatment (Chen et al. 2020a). Administering MSC-derived conditioned medium (MSC-CM) effectively reduced Iba-1 and CD4 levels, inhibited alpha-synuclein production, increased tyrosine hydroxylase levels in the striatum, and improved motor deficits in rats with rotenone-induced Parkinson’s disease. This finding highlights the potential of MSC-CM in Parkinson’s disease therapy (Chen et al. 2020c). In a PD in vitro model, MSC-generated conditioned medium effectively reduced neuroinflammation, oxidative stress, and apoptosis in MPP+-induced SH-SY5Y neuroblastoma cells (Li et al. 2019). MSC-Exos significantly reduced NLRP3-induced inflammation and cytosolic protein kinase 5 (CDK5)-related nigrostriatal autophagy; moreover, they decreased dopaminergic neuronal apoptosis and inflammation while inhibiting α-synuclein aggregation in PD mice (Li et al. 2021). MSC-Exos effectively modulate Parkinson’s disease-related neuroinflammation, reduce dopaminergic neuron apoptosis, and enhance PD motor symptoms.

### MSC-Exos and Traumatic Brain Injury

Traumatic brain injury (TBI) is a severe central nervous system disorder that is associated with high mortality and disability rates (Jacquens et al. 2022). A study revealed a significant link between TBI and neurodegenerative diseases (Brett et al. 2022). Neuroinflammation mediated by glial cell overactivation is the leading cause of brain damage secondary to TBI (Karve et al. 2016). In a study of TBI mice, after injection of MSC-Exos, TNF-α and IL-1β expression decreased. Simultaneously, inducible nitric oxide synthase (iNOS) expression was downregulated, and Arg-1 was upregulated. This manipulation shifts the conversion of microglia from proinflammatory to anti-inflammatory, thereby reducing the neuroinflammatory effects of TBI (Ni et al. 2019). After 14 days of TBI, MSC-Exos significantly reduced the size of the brain lesions. This was attributed to their role in regulating inflammation and their capacity to alleviate secondary brain damage (Ni et al. 2019). Intervention with MSC-Exos led to a decrease in activated astrocytes following TBI, and its ability to modulate inflammation was positively correlated with the duration of administration (Zhang et al. 2020). Enriched miRNA-17-92 MSC-Exos exhibit considerable promise in restoring sensory-motor and cognitive abilities in mouse models of TBI (Zhang et al. 2021). Several studies have demonstrated that the HMGB1/NF-κB pathway can be inhibited by exosomes from MSCs enriched with miR-216a-5p, thereby reducing TBI-induced neuroinflammation (Xu et al. 2020). MSC-Exos interrupt TLR4 signaling, inhibit NF-κB and MAPK phosphorylation, and mitigate neuroinflammation triggered by microglial overactivation in rats with brain injury (Thomi et al. 2019). MSC-Exos exert inhibitory neuroinflammatory and neuroprotective effects by regulating glial cells and improving the microenvironment in regions of brain injury.

### MSC-Exos and Other Neurological Disorders

MSC-Exos, a novel therapy, significantly improve cognitive function in mice suffering from sustained epilepsy (SE) (Liu et al. 2021a). By regulating glial cell activation, reducing hippocampal inflammation, and enhancing neuronal protection, MSC-Exos prevent SE-induced cognitive memory deficits and decrease cognitive activity. In a study investigating the beneficial impact of MSC-Exos on middle cerebral artery occlusion, it was discovered that MSC-Exos effectively suppressed microglial activation, simultaneously diminishing neutrophil factor and chemokine accumulation (Pathipati et al. 2021). The inhibition of MAPK11-involved astrocyte inflammation and the enhancement of antioxidant capacity are essential mechanisms by which MSC-Exos improve the prognosis of amyotrophic lateral sclerosis (ALS) (Provenzano et al. 2022). MSC-Exos exhibit immunomodulatory effects, effectively regulating microglial cell activation in multiple sclerosis (MS). Diminishing Th1 and Th17 expression enhances motility and fosters myelin regeneration (Laso-García et al. 2018). The efficacy of MSC-Exos in mitigating numerous neurological ailments is strongly connected to their function in regulating neuroinflammation, a factor that cannot be overlooked.

## Mechanisms by Which MSC-Exos Regulate Neuroinflammation

Recent research has revealed that MSC-Exos possess neuroprotective effects under neurological conditions, with neuroinflammation being a critical pathogenic mechanism. Neuroglial activation initiates neuroinflammation, prompting astrocyte activation and the release of inflammatory agents. These factors contribute to neuronal damage and exacerbate neuroinflammation (Liddelow et al. 2017; Norden et al. 2016). The regulation of glial cells has been identified as a vital link in neuroinflammation. Numerous studies have demonstrated that MSC-Exos aid in transforming microglia from a proinflammatory state to an anti-inflammatory state, suppressing activated astrocytes, and providing neuroprotection (Go et al. 2020; Garcia-Contreras and Thakor 2021; Cui et al. 2022; Xian et al. 2019). The following question then arises: how do MSC-Exos regulate glial cells?

### MSC-Exos with TLRs and NF-κB

Toll-like receptors (TLRs), including TLR2 and TLR4, serve as pattern recognition receptors (PRRs) capable of identifying pathogen-associated molecular patterns (PAMPs). TLRs are crucial in modulating CNS inflammation by regulating cytokines and chemokines, particularly in microglial activation (Sloane et al. 2010; Fiebich et al. 2018; Huang et al. 2017). CD14, a GPI-anchored protein expressed on myeloid cells, is activated by TLR4, which produces proinflammatory cytokines via MyD88-dependent and TRIF-independent signaling pathways (Ciesielska et al. 2021). Research has revealed that MSC-Exos can inhibit LPS binding to TLR4 via the TLR4/CD14 complex, manipulate IκBα and AP-1 transcription, and minimize inflammatory factor release (Thomi et al. 2019). MSC-Exos exhibit direct inhibitory effects on neuroinflammation driven by the TLR4 signaling pathway, explicitly targeting HMGB1 (Xiong et al. 2020). Additionally, research has revealed that MSC-Exos can suppress the TLR2/NF-κB signaling pathway, thereby reducing the inflammatory response by inhibiting IRAK1 expression (Zhang et al. 2022).

NF-κB, a nuclear transcription factor (Sun et al. 2022), initiates the expression of genes regulating inflammatory responses and proinflammatory factors when stimulated (Yu et al. 2020). The hippocampus of APP/PS1 mice exhibited increased NF-κB expression. However, upon treatment with MSC-Exos, TNF-α, TRAF6, and NF-κB expression decreased. This reduction attenuated the inflammatory response activated by astrocytes and mitigated the cognitive deficits characteristic of AD (Nakano et al. 2020). MSC-Exos also inhibit IRAK1 expression in astrocytes and intervene in NF-κB-mediated neuroinflammatory responses (Lai et al. 2022). Research in an LPS-induced neuroinflammation model utilizing RAW264.7 cells revealed that MSC-Exos effectively targeted tumor necrosis factor-stimulated gene-6 (TSG-6). This intervention inhibits the NF-κB/NLRP3 signaling pathway and modulates macrophage phenotypic transformation (Li et al. 2022a). NF-κB also regulates histone deacetylase 3 (HDAC3) expression, and MSC-Exos inhibit p65 phosphorylation, NF-κB transcriptional activity, HDAC3 expression, and neuroinflammation in subarachnoid hemorrhage (Lai et al. 2020). This study indicated that MSC-Exos can potentially reduce neuroinflammation and serve a neuroprotective function by hindering TLR/NF-κB activation.

### MSC-Exos with MAPK

MAPKs, as members of the serine/threonine protein kinase family, include extracellular signal-regulated kinase (ERK), c-Jun N-terminal kinase (JNK)/stress-activated protein kinase, and p38 MAPK. These kinases activate MAPK from the extracellular region to the nucleus, where they function in the immune response, cell proliferation, and apoptosis (Behl et al. 2022; Guan et al. 2020). MSC-Exos inhibit neuroinflammation in brain injury by effectively regulating LPS- and oxygen–glucose deprivation (OGD)-induced microglial activation, which occurs through suppression of the phosphorylation of P38MAPK, JNK, ERK1/2, P65, IKKαβ, and NF-κB inhibitor alpha (IKBα), ultimately decreasing the activation of these proteins (Shu et al. 2022; Chen et al. 2020b). It has been observed that MSC-Exos, which contain miR-467f and miR-466q, effectively target Map3k8 and Mk2, inhibiting p38 MAPK. This intervention has been shown to be involved in regulating neuroinflammation induced by microglial activation (Giunti et al. 2021). Research has revealed that MSC-Exos exert regulatory effects on astrocyte activation through the MAPK pathway; simultaneously, they stimulate the nuclear translocation of nuclear factor erythroid2-related Factor 2 (Nrf2), amplifying antioxidant effects and diminishing neurotoxicity (Provenzano et al. 2022). MSC-Exos exhibit anti-inflammatory and antioxidant effects by regulating neuroinflammation through MAPK signaling and synergistically collaborating with Nrf2.

### MSC-Exos with JAK/STAT

The Janus kinase/signal transducer and activator of transcription (JAK/STAT) signaling pathway is constructed of three primary elements: the tyrosine kinase-related receptor, JAK, and STAT. Upon phosphorylation by JAK, STAT converts into a dimer that permeates the nuclear membrane, ultimately substantially impacting cell survival, inflammation, and immune regulation (Khera et al. 2022; Xin et al. 2020). MSC-Exos effectively safeguard microglia from inflammatory reactions triggered by OGD stimulation. This effect is achieved by inhibiting STAT3 phosphorylation, downregulating proinflammatory factor expression, and moderating inflammatory responses (Xin et al. 2022). STAT3 activation and interaction with p38MAPK occur, while MSC-Exos reduce STAT3 and p38MAPK phosphorylation; it also inhibits the overexpression of the inflammatory mediators cyclooxygenase-2 (COX-2), monocyte chemoattractant protein-1 (MCP-1), and iNOS, leading to neuroprotective effects in subarachnoid hemorrhage (Liu et al. 2021c). Interestingly, the TBI study revealed that MSC-Exos effectively inhibited neuroinflammation by stimulating STAT3 phosphorylation, which potentially occurs because activated STAT3 further elevates IL-10 expression, initiating an autocrine feedback loop that amplifies its anti-inflammatory properties (Wen et al. 2022). However, the JAK/STAT pathway has opposite regulatory effects on various diseases, indicating that MSC-Exos could contribute to neuroinflammation by controlling the JAK/STAT pathway.

### MSC-Exos with NLRP3

The NLRP3 inflammasome, a complex consisting of NLRP3, ASC, and caspase-1, plays a crucial role in innate immunity; it regulates caspase-1-induced GSDMD-dependent pyroptosis and the release of IL-1β and IL-18. This inflammasome triggers cell death in response to infections, pathological stress, and various stimuli (Huang et al. 2021). MSC-Exos demonstrated robust potential for reducing Aβ-induced neuroinflammation while improving memory and locomotor abilities in APP/PS1 mice. The effectiveness of these agents stems from their ability to inhibit NLRP3 and caspase-1 expression (Zhai et al. 2021). In vitro studies revealed that MSC-Exos are effective inhibitors of the TSG-6/NF-κB/NLRP3 pathway, fostering the conversion of microglia to the M2 anti-inflammatory phenotype (Li et al. 2022a). MSC-Exos exhibit neuroprotective effects against ischemia‒reperfusion injury by efficiently suppressing NLRP3-induced neuronal death and regulating microglial activity (Liu et al. 2021b). Studies have shown that MSC-Exos boost FOXO3a expression, resulting in decreased inflammatory factor release and, simultaneously, decreased NLRP3, caspase-1, and GSDMD expression (Hu et al. 2021). Furthermore, MSC-Exos exhibit a more significant inhibitory effect on NLRP3 under hypoxic pretreatment (Kang et al. 2021). MSC-Exos robustly inhibited PDCD4 expression, thereby restraining NLRP3 and inhibiting inflammatory factors. This process mitigates neuroinflammation and brain damage after cerebral hemorrhage (Ding et al. 2021). MSC-Exos exhibit excellent potential to attenuate neuroinflammation by modulating NLRP3 inflammatory vesicles.

### Others:

(1) BACE1, an Aβ precursor protein cleaving enzyme in microglia, exacerbates AD by promoting Aβ production and inflammatory responses (Singh et al. 2022). Nonetheless, MSC-EVs that deliver miR-29c-3p can potentially inhibit BACE1, mitigate Aβ accumulation, and ameliorate neuroinflammation and neuronal apoptosis in AD mouse models. This efficacy is derived from activating the Wnt/β-catenin pathway (Sha et al. 2021). (2) Neutrophil gelatinase-associated lipid transport protein 2 (LCN2), secreted by activated astrocytes, serves as a critical mediator of neuroinflammation and neurodegeneration (Kim et al. 2022), and MSC-Exos containing miR-138-5p effectively downregulate LCN2 and inhibit neuroinflammation induced by astrocyte activation (Deng et al. 2019).

**Fig. 2:**
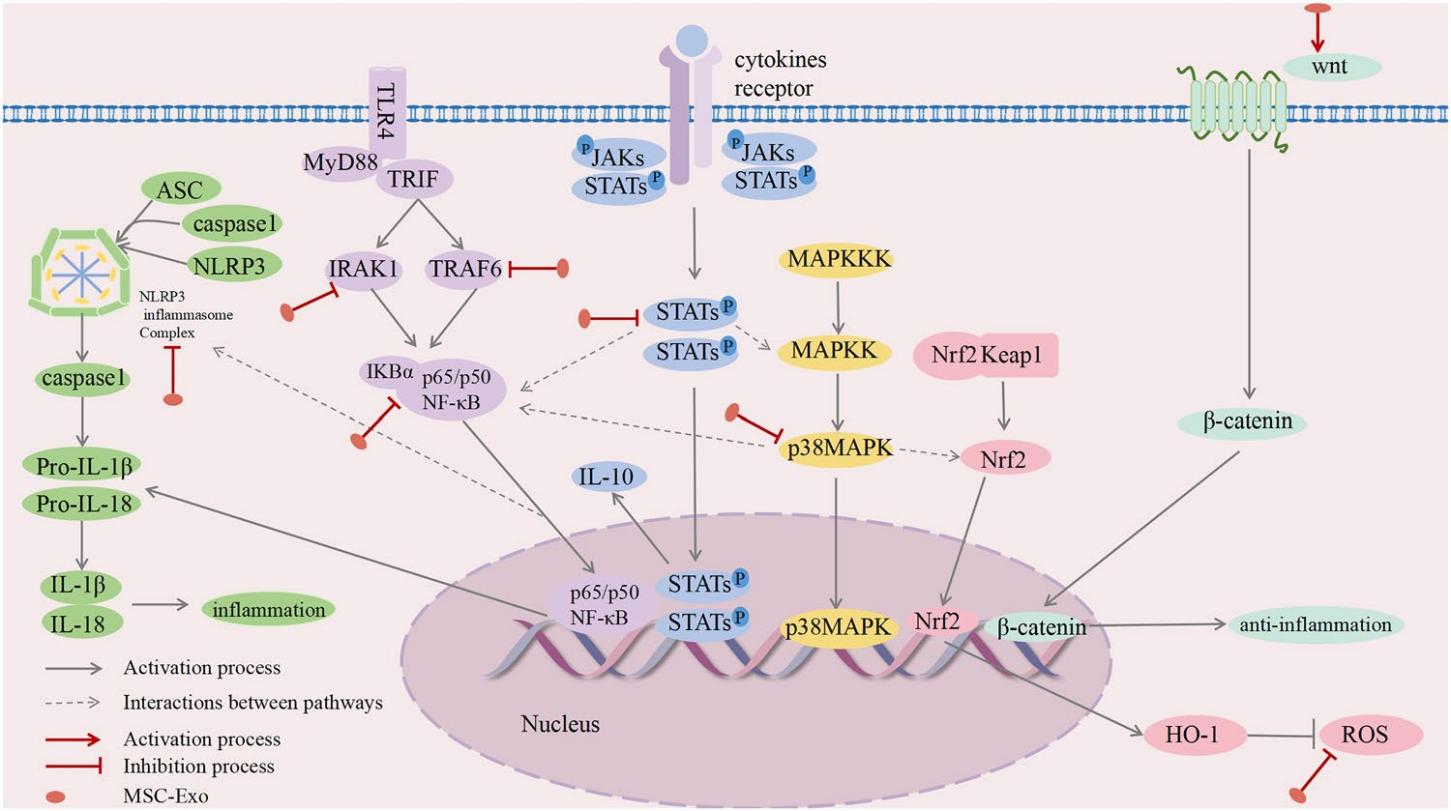
MSC-Exos exert neuroprotective effects mainly by modulating the TLR, NF-ĸB, MAPK, JAK/STAT, NLRP3, and Wnt/β-catenin pathways, thereby improving neuroinflammation

In essence, the genesis and progression of neuroinflammation rely heavily on signaling pathways. These pathways are interconnected, forming a regulatory network that influences one another. MSC-Exos can potentially alleviate neuroinflammation by adjusting these signaling pathways, opening up a new avenue for treating neurological disorders (Fig. 2).

## MSC-Exos Carry miRNAs that Play a Role in Neuroinflammation

MSC-Exos, abundant in miRNAs, significantly contribute to cellular regulation (Schulz-Siegmund and Aigner 2021). These 22-nucleotide noncoding RNA molecules bind to the 30 untranslated regions (UTRs) or open reading frames (ORFs of target mRNAs, dictating mRNA degradation or translation inhibition, ultimately affecting protein expression (Das and Rao 2022). Research has demonstrated that MSC-Exos carry miRNAs capable of affecting CNS disorders (Iranifar et al. 2019). miR-216a-5p (Xu et al. 2020), miR-193b-3p (Lai et al. 2020), miR-21a-5p (Xin et al. 2022), miR-26b-5p (Liu et al. 2021c), miR-181b (Wen et al. 2022), and miR-183-5p (Ding et al. 2021) trigger the transformation of microglia into an anti-inflammatory state, diminish proinflammatory factors, and thereby achieve neuroprotective outcomes in acute CNS injuries. MSC-Exos, which are enriched in miRNA-17-92, effectively promote the recovery of sensory-motor and cognitive functions in TBI mice compared to those in mice not loaded with exosomes (Zhang et al. 2021). MiR-22 (Zhai et al. 2021) can regulate GSDMD-induced focal death, inhibit inflammation, and improve AD motor and memory abilities. Moreover, a study confirmed that MSC-Exos containing miR-146a (Nakano et al. 2020), miR-21 (Cui et al. 2018), and miR-29c-3p (Sha et al. 2021) interfered with neuroinflammation induced by Aβ stimulation and attenuated neuronal apoptosis. In addition, MSC-Exos enriched with miR-188-3p can inhibit inflammatory vesicles and ameliorate PD nigrostriatal dopamine neuronal damage by suppressing excessive autophagy (Li et al. 2021). In ALS studies, miR-466q and miR-467f in MSC-Exos were shown to downregulate Mapk11, miR-466 m-5p, and miR-466i-3p to promote the nuclear translocation of Nrf2, and miRNAs regulate inflammatory responses and oxidative stress in astrocytes through anti-inflammatory and antioxidant activities (Provenzano et al. 2022). Moreover, the role of miR-138-5p in astrocyte activation-mediated neuroinflammation has been identified (Deng et al. 2019). Taken together, these findings indicate that miRNA-rich MSC-Exos play a significant role in regulating neuroinflammation (Table 2).

**Table 2:** MSC-Exos carry different miRNAs that play neuroprotective roles in neurological diseases

Disease	miRNA
TBI	miRNA-17-92 Zhang et al. (2021), miR-216a-5p Xu et al. (2020), miR-193b-3p Lai et al. (2020), miR-21a-5p Xin et al. (2022), miR-26b-5p Liu et al. (2021c), miR-181b Wen et al. (2022), miR-183-5p Ding et al. (2021)
AD	miR-22 Zhai et al. (2021), miR-146a Nakano et al. (2020), miR-21 Cui et al. (2018), miR-29c-3p Sha et al. (2021)
PD	miR-188-3p Li et al. (2021)
ALS	miR-466q, miR-467f, miR-466 m-5p, and miR-466i-3p Provenzano et al. (2022)

## Conclusion

MSC-Exos exhibit low immunogenicity and efficiently cross the BBB, allowing them to reach lesion sites easily. The miRNAs they carry influence the differentiation of glial cells and regulate neuroinflammation through signaling pathways. This process triggers the release of cytokines and inflammatory mediators, enhances apoptosis resistance, and has neuroprotective effects. Uncovering the mechanism of MSC-Exos in neurological diseases is thus crucial. Previous studies have shown that various components of MSC-Exos might collaborate to mitigate neuroinflammation through multiple cellular processes.

Nonetheless, the specific role of each MSC-Exo component in reducing neuroinflammation remains to be elucidated. Enhancing the targeting efficiency of MSC-Exos is also a crucial aspect to consider for future research. Despite their broad potential in treating neurological disease, MSC-Exos face several challenges; these include overcoming the barriers associated with MSC-Exo extraction technology, establishing standardized quality control measures, and optimizing the clinical benefits of these materials. Furthermore, few clinical studies on MSC-Exos exist, necessitating numerous basic and clinical investigations to unravel the underlying mechanism of MSC-Exos, particularly their role in neuroinflammation regulation and clinical safety, which will ultimately facilitate the early use of MSC-Exos in neurological disease treatment.

## Data Availability

Not applicable.
